# Inhibition of calpain-1 stabilizes TCF11/Nrf1 but does not affect its activation in response to proteasome inhibition

**DOI:** 10.1042/BSR20180393

**Published:** 2018-09-19

**Authors:** Karolin Nowak, Ramona M. Taubert, Stefanie Haberecht, Simone Venz, Elke Krüger

**Affiliations:** 1 Institute of Biochemistry, Charité – Universitätsmedizin Berlin, Charitéplatz 1, 10117 Berlin, Germany; 2 Institute of Medical Biochemistry and Molecular Biology, Universitätsmedizin Greifswald, Klinikum DZ7, Ferdinand-Sauerbruch-Str., 17475 Greifswald, Germany

**Keywords:** calpains, proteostasis, proteasomes, TCF11/Nrf1

## Abstract

Protein degradation is essential to compensate for the damaging effects of proteotoxic stress. To ensure protein and redox homeostasis in response to proteasome inhibition, the cleavage and nuclear translocation of the endoplasmic reticulum (ER)-bound transcription factor TCF11/Nrf1 (*NFE2L1*) is crucial for the activation of rescue factors including the synthesis of new proteasomal subunits. Even though TCF11/Nrf1 is an essential transcription factor, the exact mechanisms by which it is activated and stabilized are not fully understood. It was previously shown that the calcium-dependent protease calpain-1 interacts with TCF11/Nrf1 and the TCF11/Nrf1 cleavage site is a potential calpain target. Here, we tested the hypothesis that calpain-1 or -2 cleave TCF11/Nrf1. However, we did not find a role for calpain-1 or -2 in the activation of TCF11/Nrf1 after proteasome inhibition neither by using chemical inhibitors nor siRNA-mediated knockdown or overexpression of calpain subunits. Instead, we found that TCF11/Nrf1 is digested by calpain-1 *in vitro* and that calpain-1 inhibition slows down the degradation of membrane-bound TCF11/Nrf1 by the proteasome in cultured cells. Thus, we provide evidence that calpain-1 is involved in the degradation of TCF11/Nrf1. Furthermore, we confirmed DDI2 as an essential factor for TCF11/Nrf1 activation and demonstrate an undefined role of DDI2 and calpain-1 in TCF11/Nrf1 stability.

## Introduction

Proteostasis describes the balance of biogenesis, folding, trafficking and degradation of proteins within cells and their surroundings. Loss of its maintenance is associated with different degenerative disorders such as Alzheimer’s disease and various types of cancer. Therefore, it is essential to compensate for the damaging effects of proteotoxic and redox stress by different mechanisms such as the removal of misfolded and damaged proteins. One essential component of the proteostatis network is the ubiquitin proteasome system (UPS) [1,2].

Previously, the invers coupling of protein and redox homeostasis was demonstrated for endoplasmic reticulum (ER) and cytoplasmic proteins [3]. A transcriptional feedback loop links UPS-dependent protein degradation to different stress responses via the activation of the ER-bound transcription factor TCF11/Nrf1, which belongs to the Cap‘n’Collar family. The *NFE2L1* gene encodes different isoforms of this transcription factor. The most important ones are the large form known as TCF11 and the slightly smaller form Nrf1. In humans, both isoforms are expressed whereas mice only express the smaller Nrf1 isoform. Thus, in human cells cleavage of the TCF11 and Nrf1 isoforms results in four detectable proteins of different size [4].

TCF11/Nrf1 usually resides in the ER-membrane from where it is extracted by p97, ubiquitinylated with the help of HRD1 and rapidly degraded by the proteasome as part of the ER-associated degradation system (ERAD). Its half-life is ~10 min with slight variations depending on cell type [4–6]. Upon proteasome inhibition, TCF11/Nrf1 is stabilized, cleaved and translocates into the nucleus where it can activate its target genes, such as genes encoding the 26S proteasome subunits, components of the ubiquitin conjugation and deconjugation machinery as well as genes involved in oxidative-stress responses. However, little is known about the mechanism of TCF11/Nrf1 activation, its cleavage and transport into the nucleus [6]. Recently, DDI2 was described as the protease involved in cleavage of TCF11/Nrf1 leading to its activation. However, it remains unknown whether DDI2 directly cleaves TCF11/Nrf1 or whether it activates another protease responsible for TCF11/Nrf1 cleavage [7].

Before the identification of DDI2, there was a long-standing debate about the cleaving protease, with the proteasome and calpain-1 being the two candidates most often cited [8,9]. By now, it has been shown that the proteasome is not involved in this process [10].

However, there is still evidence that calpain-1 may be involved in this process as a yeast-two-hybrid assay using a human heart cDNA library could show the interaction between calpain-1 and TCF11/Nrf1 and the transcription factor has a potential calpain-1 cleavage site in the region where it is cleaved to induce its activation [11].

The calpain family consists of 15 different isoforms with the µ- and m-calpain – or calpain-1 and -2 – being the most common forms in mammalia. They are activated by calcium concentrations in the micromolar range and are involved in cytoskeletal remodelling, cellular differentiation and apoptosis. Calpain-1 and -2 are heterodimers composed of a shared small regulatory subunit CAPNS1 (29 kDa) associated with either calpain-1 or -2, which is essential for calpain-1 and -2 activity. The distinct large catalytic subunits are encoded by the genes CAPN1 and CAPN2 (encoding subunits of 80 kDa) [12].

Due to the previous evidence of calpain-1 binding to TCF11/Nrf1 and the potential cleavage site, we followed the hypothesis that calpain-1 or -2 is the protease, which cleaves TCF11/Nrf1. So far, no experimental evidence has been published to proof, whether calpain-1 or -2 might be involved in activation of TCF11/Nrf1 at the ER or not. Here, we did not find any evidence of an involvement of calpain-1/2 in the activation of TCF11/Nrf1 after proteasome inhibition. In contrast, we provide evidence that calpain-1 is involved in the degradation of membrane-bound TCF11/Nrf1 under non-inducing conditions. In addition, we confirmed that DDI2 is necessary for the TCF11/Nrf1 cleavage.

## Materials and methods

### Cell culture

For all experiments, Ea.Hy-926 (ATCC^®^ CRL-2922™), a human endothelial cell line, or their lysates were used. Cells were grown in Basal Iscove Medium supplemented with 10% FCS and penicillin and streptomycin.

### Chemical inhibition of calpain

Cells were pre-treated with different concentrations of PD150606 (Calbiochem) or PD151746 (Abcam) or BAPTA-AM (Abcam) for 1 h before being treated with Bortezomib (BTZ, Velcade^®^, clinical grade) for the indicated time points. For experiments without BTZ, cells were treated with the calpain inhibitors for the indicated time points. The efficacy of BAPTA-AM was verified using a fluorescence-based method with Fluo-4-AM to detect intracellular calcium and flow cytometry as a read-out. The calcium complexed by BAPTA is maintained in the cell using probenecid. Fluo-4-AM has a higher affinity for calcium than BAPTA-AM.

### Immunoblots

Ea.Hy-926 cell pellets were directly lysed in 2× SDS sample buffer and then incubated at 95°C for 10 min to ensure complete lysis. Lysates were separated on 6.5–15% gradient cells and transferred to PVDF membranes. Membranes were blocked in 5% milk and incubated overnight with the respective primary antibodies. The following day, membranes were washed, incubated with secondary antibodies, washed again and developed using ECL.

Primary antibodies used were: GAPDH (polyclonal rabbit (pcRb), Santa Cruz Biotechnology), β-Aktin (monoclonal mouse (mcM), C4, Santa Cruz Biotechnology), TCF11/Nrf1 (mcRb, D5B10, Cell Signalling), DDI2 (pcRb, ab197081, Abcam), p97/VCP (pcRb, Lab Stock), PSMA7/α4 (pcRb, Lab Stock), PSMA1/α6 (mcM, MCP20, Enzo Life Sciences), Tubulin (mcM, Covance Antibody Products), Calnexin (mcM, BD Biosciences), Lamin B1 (mcM, Invitrogen), CAPNS1 (pcRb, GeneTex). Secondary antibodies used were anti-mouse HRP and anti-rabbit HRP (Calbiochem).

### Nuclear fractionation

To separate nuclear and non-nuclear proteins, cells were resuspended in buffer A (10 mM HEPES, pH 7.82; 10 mM KCl; 0.1 mM EDTA) and incubated for 20 min on ice. NP-40 to 0.25% (v/v) was added for 3 min and the nuclei pelleted by centrifugation for 10 min at 5000×***g***, 4°C. The supernatant contained the non-nuclear proteins. The nuclei were lysed using puffer B (20 mM HEPES pH 7.82; 500 mM KCl; 1 mM EDTA; 1 mM DTT; 10% Glycerin) for 30 min on ice. Genomic DNA and cell fragments were removed by centrifugation for 10 min at 12000×***g***, 4°C.

### Cycloheximide decay experiments

For the decay experiments, the medium was removed and replaced with fresh medium containing 100 µg/ml cycloheximide. The cells were lysed directly in 2× sample buffer after 10, 20 or 30 min. For the control, cells were left untreated.

### *In vitro* digestion with calpain-1

To digest TCF11/Nrf1 *in vitro*, the membrane fraction was isolated from cell pellets. First, pellets were resuspended in buffer 1 (50 mM Tris, pH 7.7; 50 mM NaCl; 5 mM MgCl_2_) and the cells were lysed by three freeze–thaw cycles in liquid nitrogen. The cytosolic fraction was removed by centrifugation for 10 min at 1000×***g***, 4°C. The remaining pellet was resuspended in buffer 2 (buffer 1 + 1% NP-40) and incubated on ice for 30 min, followed by centrifugation for 10 min at 6000×***g***, 4°C. The supernatant contained the membrane fraction, which was used for the digestion.

For the digestion, the components of the Calpain Activity Assay Kit (ab65308, Abcam) were used. To the isolated membrane fraction, the Reaction Buffer and Active Calpain I as well as different inhibitors such as the impermeant Calpain Inhibitor Z-LLY-FML from the Calpain Activity Assay Kit, BTZ or NMS-873 (SelleckChem) were added. The mix was incubated for 30 min at 37°C before sample buffer was added to the sample and it was heated for 10 min to 95°C.

For the digestion using the proteasome, the membrane fraction was mixed with proteasome test buffer (50 µM Tris, 5 µM MgCl_2_ and 2 µM ATP) and 0.5 µg proteasome isolated from human erythrocytes were added. The mix was also incubated for 30 min at 37°C before adding the sample buffer and heating the samples to 95°C for 5 min.

### Fluorescent microscopy

Cells were seeded onto coverslips and treated the following day. After the treatment had ended, cells were fixed in 4% formalin/PBS for 20 min at room temperature, permeabilised for 10 min using 0.1% triton-X100 in PBS and blocked in 3% FCS for 30 min. Primary antibodies were incubated at 4°C overnight in 3% FCS, coverslips were washed three times with PBS and then secondary antibody were incubated 30 min at room temperature. Coverslips were washed again, incubated with DAPI and then mounted onto slides for fluorescent microscopy.

Primary antibody: TCF11/Nrf1 (mcRB, D5B10, Cell Signalling); secondary antibody: goat anti-rabbit Alexa-488 (LifeTechnologies).

### Transfection with plasmid-DNA

Ea.Hy-926 cells were transfected with plasmid DNA using Lipofectamin 2000 (Thermo Fisher Scientific), following the manufacturer’s manual. Nrf1-3xFlag (RDB-2867) and Nrf1(m1)-3xFlag (RDB-2868) were a kind gift from R.J. Deshaies (Radhakrishnan et al. [6]). Control plasmid pcDNA3.1 was from Invitrogen and TCF11-V5 was previously cloned in the lab. The flag-tagged CAPN1 (Addgene #60941) and CAPN2 (Addgene #60942) have been previously described [13].

### siRNA transfection

siRNA transfection was carried out using HiPerfect, following the manufacturer’s protocol using 40 nM CAPNS1 (siRNA _ s2385, LifeTechnologies) for 48 h before treatment.

### RNA isolation and cDNA synthesis

RNA was isolated using the High Pure RNA Isolation Kit (Roche) following the manufacturer’s instructions. cDNA synthesis was performed with the Transcriptor High Fidelity cDNA Synthesis Kit (Roche), following the manufacturer’s protocol using 500 ng RNA.

### Quantitative real-time PCR

Quantitative real-time PCR (qRT-PCR) was carried out using TaqMan^®^ Gene Expression Assays and the TaqMan^®^ Universal PCR Master Mix (Applied Biosystems) following the manufacturer’s protocol. The relative gene expression was calculated using the ΔΔ*C*_T_ method and normalised against hypoxanthine phosphoribosyltransferase1 (*HPRT1*).

The following master mixes were used: *HPRT1* (Hs99999909_m1), *PSMB6* (Hs00382586_m1), *PSMC4* (Hs00197826_m1) and *PSMA3* (Hs00541061_m1).

### Knockout using CRISPR–Cas

DDI2 knockout clones were generated using the CRISPR–Cas technique following established protocols with the pSpCas9(BB)-2A-Puro (PX459) plasmid (Addgene #62988) [14]. The sequence for the guide RNA was previously published [7].

## Results

### Chemical inhibition of calpain-1/2 does not alter TCF11/Nrf1 processing and nuclear translocation

TCF11/Nrf1 is cleaved before it can translocate into the nucleus where it acts as a transcription factor [5]. It has been hypothesised that calpain-1 is involved in its activation. To analyse the effect of calpain-1 and -2 on the activation of TCF11/Nrf1, various inhibitors were used to block its activity – two calpain-1/2 inhibitors and a calcium chelator. PD150606 is a specific calpain-1/2 inhibitor with a *K*_i_ value of 210 nM for calpain-1 and 370 nM for calpain-2 [15]. The second inhibitor used – PD151746 – has a 20-fold higher selectivity for calpain-1 (*K*_i_ 260 nM) than for calpain-2 (5.33 µM). In fact, PD151746 cannot completely inhibit calpain-2 even at very high concentrations [15], and it can therefore be seen as a specific calpain-1 inhibitor. In addition, we also used the cell-permeable calcium chelator BAPTA-AM as the activity of calpain-1/2 is dependent on intracellular calcium levels.

**Figure 1:**
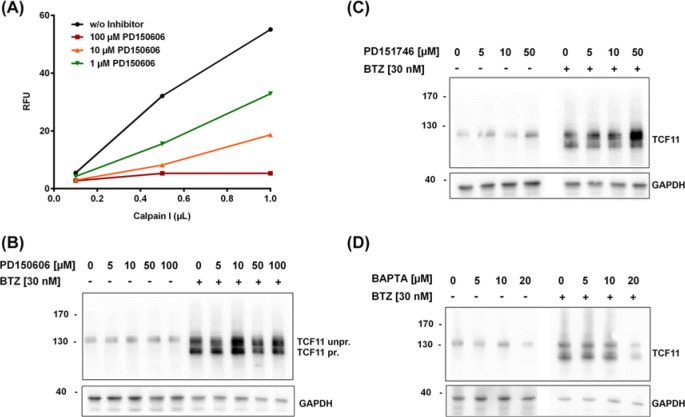
TCF11 processing after chemical inhibition of calpain-1/2 followed by long-term inhibition of the proteasome (**A**) *In vitro* testing of PD150606 using recombinant calpain-1 in different concentrations, inhibition was carried out for 1 h at 37°C, and calpain-1 activity was measured using the fluorescent substrate Ac-LLY-AFC. (**B–D**) Ea.Hy-926 cells were pretreated for 1 h with different inhibitors and then treated or not with 30 nM of the proteasome inhibitor BTZ for 16 h, total cell lysates were analysed by immunoblot; (B) PD150606 and (C) PD151746 inhibition in indicated concentrations, (D) inhibition using the calcium chelator BAPTA-AM, representative figures from *n*=3 each.

To determine the correct concentration for our experiments, we first tested the inhibiting capacity of PD150606 *in vitro* with recombinant active calpain-1 using a fluorescent substrate. We found that at concentrations of 100 µM PD150606 calpain-1 was completely inhibited. Even when very high calpain-1 concentrations were used, we could no longer detect any fluorescent signal above background (Figure 1A). Using 10 µM PD150606, we saw a remaining activity of calpain-1 of ~30% using the highest starting concentration of calpain-1 *in vitro*. In contrast, concentrations as low as 1 µM were not sufficient to effectively block calpain-1 activity (Figure 1A). We also tried to directly test calpain activity within the cells; however, this proved to be difficult as most available calpain substrates are not cell permeable or are unspecific and also cleaved by the proteasome.

Therefore, we decided to use a range of different concentrations of calpain inhibitors (between 5 and 100 µM) in the cell experiments to test the viability of cells after 4 h of inhibitor treatment. For both inhibitors (PD150606, PD151746) concentrations up to 50 µM were well tolerated; however, the viability of cells decreased starting from 50 µM inhibitor concentration (data not shown). This phenomenon was even more apparent when PD151746 was used together with BTZ (no cell sample could be obtained anymore for 100 µM PD151746 together with BTZ). With the intracellular calcium chelator BAPTA-AM cells started to detach from the tissue culture plate and could not be used for experiments anymore at concentrations over 20 µM (data not shown).

Based on our starting experiments, we used the calpain inhibitor PD150606 in a range of 5–100 µM, PD151746 from 5 to 50 µM and the calcium-chelator BAPTA-AM in concentrations between 5 and 20 µM. The efficacy of BAPTA-AM as a calcium-chelator was demonstrated using Fluo-4-AM by flow cytometry showing an increase of complexed calcium in the presence of BAPTA-AM (Supplementary Figure S1B).

Next, we analysed the TCF11/Nrf1 activating cleavage in Ea.Hy-926 cells that were pre-treated with calpain-1/2 inhibitors or BAPTA-AM before inducing TCF11/Nrf1 activation by inhibition of the proteasome using low concentrations of BTZ overnight. Using the inhibitors in combination with BTZ treatment, no differences were observed in the cleavage of TCF11/Nrf1 with any of the concentrations or inhibitors used (Figure 1B–D and Supplementary Figure S1A): in all samples TCF11/Nrf1 was still cleaved, which can be seen by the appearance of a second double band in BTZ-treated samples compared with the untreated samples. However, densitometric analysis of samples after calpain-1/2 inhibition using PD150606 revealed more total levels of unprocessed and processed TCF11/Nrf1 (Figure 1B,C and Supplementary Figure S1A,C). Strikingly, treatment with the calpain-1 specific inhibitor PD151746 resulted in an increase in unprocessed TCF11/Nrf1, whereas the processed form was less affected (Figure 1C; Supplementary Figure S1A). Similar results were obtained when higher concentrations of BTZ (1 µM) were used for 3 h (data not shown).

**Figure 2:**
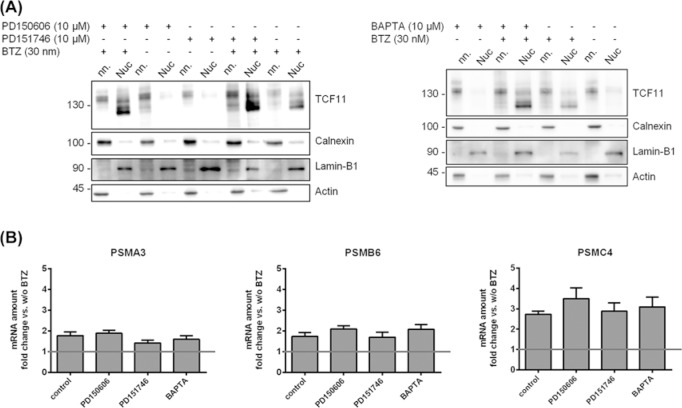
TCF11 localisation and activation of target genes after chemical inhibition of calpain-1/2 followed by proteasome inhibition Ea.Hy-926 cells were treated for 1 h with 10 µM of the calpain inhibitors PD150606, PD151746 and BAPTA-AM, and then treated or not with 30 nM BTZ for 16 h. (**A**) Immunoblot: Cell pellets were separated into nuclear (Nuc) and non-nuclear fractions (nn) and analysed for TCF11/Nrf1 expression and Calnexin, Actin and Lamin-B1 as controls, representative figures from *n*=3. (**B**) qRT-PCR: RNA was isolated and used to analyse expression of *PSMA3, PSMB6* and *PSMC4*, values were calculated using the ΔΔ*C*_T_ method with *HPRT-1* as housekeeping gene. The samples were normalised to samples without BTZ treatment (set to 1), mean ± SEM, *n*=4.

Next, we analysed changes in TCF11/Nrf1 location by cell fractionation and by immunofluorescence microscopy because activation of TCF11/Nrf1 is accompanied by its nuclear translocation [5]. As we found no difference in the processing of TCF11 comparing high and low calpain-1/2 inhibitor concentrations, we decided to use 10 µM for the next experiments, because of the higher cell viability. Without BTZ treatment, TCF11/Nrf1 was only detected in the non-nuclear fraction and located at the membrane, respectively (Figure 2A and Supplementary Figure S2). After proteasome inhibition using BTZ, a translocation of TCF11/Nrf1 into the nucleus was observed in all samples (Figure 2A and Supplementary Figure S2) with a slight increase in the amount of nuclear TCF11/Nrf1 observed in samples treated with calpain-1/2 inhibitors and BAPTA compared with the control. However, the translocation itself was not affected by calpain inhibition.

The activation of TCF11/Nrf1 by proteasome inhibition induces the *de novo* expression of proteasomal subunits [5]. Therefore, we analysed mRNA levels of *PSMA3, PSMB6* and *PSMC4* by qRT-PCR with and without proteasome inhibition following calpain-1/2 inhibition. For all three proteasomal subunits, a significant increase after BTZ treatment compared with the untreated control was observed (Figure 2B). In contrast, inhibition of calpain-1/2 did not cause significant differences in the up-regulation of the mRNA of the proteasomal subunit following proteasome inhibition (Figure 2B).

In conclusion, using chemical inhibition of calpain-1/2, no significant differences in TCF11/Nrf1 cleavage were observed, whereas its nuclear translocation or activation of its target genes was observed.

### Knockdown of the shared small subunit of calpain-1/2

**Figure 3:**
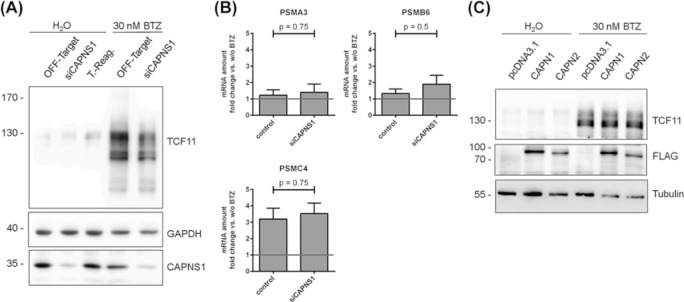
Knockdown of the regulatory subunit CAPNS1 and overexpression of CAPN1 and CAPN2 (**A** and **B**) Ea.Hy-926 cells were transfected with siRNA against CAPNS1 or an OFF-Target siRNA. (A) Twenty-four hours later cells were treated with 30 nM BTZ for 16 h, total cell lysates were analysed by immunoblot, representative image from *n*=3, (B) RNA was isolated and used for qRT-PCR to analyse expression of *PSMA3, PSMB6* and *PSMC4*, values were calcuated using the ΔΔ*C*_T_ method with *HPRT-1* as housekeeping gene and the normalised to samples without BTZ treatment (set to 1), mean ± SEM, *n*=3. (**C**) Ea.Hy-926 cells were transfected with flag-tagged constructs for the large subunits of calpain-1 (CAPN1) and -2 (CAPN2). The following day, the cells were treated with 30 nM BTZ for 16 h, total cell lysates were analysed by immunoblot, representative image from *n*=3.

To verify the results obtained using chemical inhibition of calpain-1/2, we decided to silence the expression of the shared small subunit calpain S1, which is necessary for the activity of both calpain-1 and -2 using siRNA. To this end, Ea.Hy-926 cells were transfected with an siRNA cocktail against calpain S1 and knockdown was confirmed by immunoblot (Figure 3A). Ea.Hy-926 cells were then treated with BTZ. Calpain S1 knockdown did not lead to any changes in the cleavage of TCF11/Nrf1 after BTZ treatment (Figure 3A) as the same TCF11/Nrf1 profile was detected via immunoblot analysis. We also isolated RNA from these siRNA transfected cells after BTZ treatment, which was used for qRT-PCR. There were no significant differences between control siRNA and calpain S1 siRNA transfected Ea.Hy-926 cells in the induction of *PSMA3, PSMB6* and *PSMC4* after BTZ treatment (Figure 3B) suggesting that calpain-1/2 together with its regulatory subunit calpain S1 do not substantially impact TCF11/Nrf1 activation.

### Overexpression of the larger subunits of calpain-1 and -2

Chemical inhibition of calpain-1/2 and knockdown of calpain S1 did not show any effects on the processing and activation of TCF11/Nrf1. We next tested whether overexpression of calpain-1/2 could enhance the cleavage and activation of TCF11/Nrf1 in comparison with the empty vector control pcDNA3.1.

Ea.Hy-926 cells were transfected with different FLAG-tagged constructs expressing calpain-1 or -2 and treated them with BTZ for 16 h the following day. Total lysates of the samples were analysed by immunoblot (Figure 3C). Expression of the calpain constructs was verified by the presence of the FLAG signal at ∼80 kDa. Furthermore, the blot showed stabilization and cleavage of TCF11/Nrf1 upon BTZ treatment but no differences were observed between the cells transfected with the calpain constructs and the pcDNA3.1 control transfected Ea.Hy-926 cells. To exclude cell-type specific effects, we repeated this transfection experiment in HEK293 cells. In HEK293 cells transfected with either calpain-1 or calpain-2, we could not observe any difference for TCF11/Nrf1 cleavage and activation in response to BTZ (Supplementary Figure S3) similar to the Ea.Hy-926 transfection experiments. In this experiment, again, no impact of calpain on TCF11/Nrf1 cleavage and activation could be observed.

### Calpain-1 digests TCF11/Nrf1 *in vitro*

**Figure 4:**
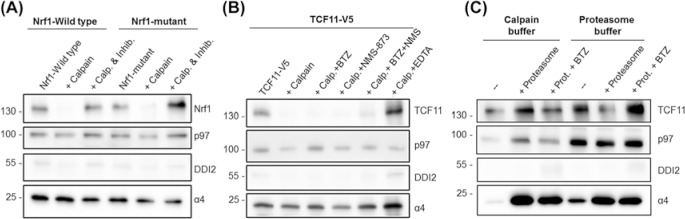
Digestion of cellular membrane fraction with calpain-1 or 26S proteasome Cells were transfected with wild type-Nrf1 (WT), a mutant Nrf1 version which cannot be cleaved anymore (**A**) or a TCF11-V5 construct (**B** and **C**). Twenty four hours after transfection, cells were harvested and the membrane fraction was isolated. (A) Calpain reaction buffer, calpain-1 and the impermeant calpain inhibitor Z-LLY-FML (&Inhib.) were added to the isolated membrane fraction and incubated for 30 min at 37°C. Samples were analysed by immunoblot for expression of Nrf1, p97, DDI2 and α4. (B) Calpain reaction buffer as well as 1 µM of the proteasome inhibitor BTZ (+BTZ) and/or 10 µM of the p97-inhibitor NMS-873 (+NMS-873) or 5 µM EDTA (+EDTA) were added to the isolated membrane fraction. The sample was incubated for 30 min at 37°C before calpain-1 was added and further incubated at 37°C for 20 min. (C) Calpain reaction buffer or proteasome digestion buffer were added to the isolated membrane fraction. Isolated 26S proteasome and 1 µM BTZ were added. The sample was incubated for 30 min at 37°C.

After confirming that chemical inhibition had no effect on the activation of TCF11/Nrf1, next calpain-1’s effect on TCF11/Nrf1 was tested *in vitro*. Ea.Hy-926 cells were transfected with either TCF11-V5 or flag-tagged Nrf1-WT and cleavage resistant Nrf1-mutant constructs, which cannot be cleaved for activation (Supplementary Figure S4A) [6]. The transfected cells were harvested and the membrane fraction isolated as TCF11/Nrf1 is a membrane protein. The isolated membrane fraction with enriched TCF11/Nrf1 was then digested with recombinant calpain-1 (the same as was used in inhibitor testing, compare with Figure 1A). The addition of calpain-1 led to complete digestion (but not cleavage) of both the wild type and mutant Nrf1 protein, which was inhibited using the calpain inhibitor Z-LLY-FML (Figure 4A and Supplementary Figure S4B). Irrespective of the activating cleavage site calpain-1 was able to process Nrf1 to lower molecular weight fragments in the size of 55, 40, 35 and 25 kDa, but did not activate Nrf1 by limited proteolysis (Supplementary Figure S4B). The same effect was observed using lysates from cells transfected with the TCF11-V5 construct and untransfected cells where only endogenous TCF11/Nrf1 was present (data not shown). As expected p97 and the proteasome subunit α4 as components of the ERAD system were also present in the membrane fraction [5–10]. Thus, the role of the proteasome and p97 in the calpain-mediated digestion of TCF11/Nrf1 were analysed in preparations from TCF11-V5 transfected cells. However, neither inhibition of the proteasome using BTZ nor inhibition of p97 using NMS-873 had an effect on the digestion of TCF11/Nrf1 by calpain-1. Only the calcium chelator EDTA could stop the digestion (Figure 4B).

This appeared to be in contrast with published data showing that TCF11/Nrf1 is degraded by the proteasome as part of the ERAD system [5]. However, the calpain digestion buffer might not allow for proteasomal degradation. Therefore, digestion using purified 26S proteasome was tested using calpain digestion buffer and a buffer typically used for proteasome activity measurements (‘proteasome digestion buffer’). Proteolytic degradation of TCF11/Nrf1 by the 26S proteasome was only observed in the proteasome digestion buffer. The proteasome is inactive in the calpain digestion buffer (Figure 4C). This indicates that calpain-1 is able to digest membrane-bound TCF11/Nrf1 *in vitro* independent of the proteasome and independent of the previously determined cleavage site.

### Inhibition of calpain-1 or -2 delays degradation of TCF11/Nrf1

**Figure 5:**
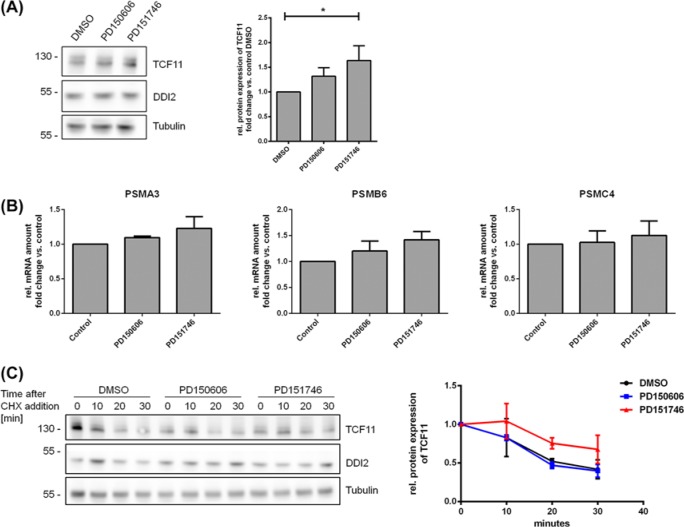
Expression of TCF11 and proteasomal subunits after treatment with calpain inhibitors (**A**) Ea.Hy-926 cells were treated with 10 µM of the calpain inhibitors PD150606 and PD151746 for 4 h, cells were lysed and analysed for the expression of TCF11 and Tubulin by immunoblot, bands were analysed by densitometry and normalised to the untreated control, *n*=6. (**B**) Ea.Hy-926 cells were treated with 10 µM of the calpain inhibitors PD150606 and PD151746 for 18 h, RNA was isolated, analysed by qRT-PCR. Expression was normalised for the house-keeping gene *HPRT-1* and the untreated control sample, *n*=4. (**C**) Ea.Hy-926 cells were treated with 25 µM of the calpain inhibitors PD150606 and PD151746 for 4 h, then 100 µg/ml cycloheximid was added to inhibit translation. Cells were lysed at indicated time points after adding cycloheximid and analysed for the expression of TCF11/Nrf1 and Tubulin. Bands were analysed by densitometry and normalised to Tubulin and the sample at time point 0, *n*=3.

After observing that inhibition of calpain-1 affected the amount of uncleaved but not cleaved TCF11/Nrf1 and that calpain-1 was able to degrade TCF11/Nrf1 *in vitro*, we concluded that calpain-1 may be involved in the degradation and the stability of this transcription factor. Consequently, we analysed the impact of calpain-1/2 on the stability of TCF11/Nrf1 in cells. For these experiments, the proteasome was not inhibited as we ruled out in the previous experiments that calpain-1/2 have an effect on the cleavage and activation of TCF11/Nrf1. First, we analysed TCF11/Nrf1 protein levels using immunoblot. Inhibition of calpain-1/2 lead to increased levels of TCF11/Nrf1 protein, which was significant for the inhibitor PD151746 (Figure 5A). Next, we tested whether calpain-1/2 inhibition alone has an effect on the transcription of new proteasomal subunits. We found no significant effect of using calpain-1/2 inhibitors on the expression of the proteasomal subunits *PSMA3, PSMB6* and *PSMC4*; however, a slight but not significant increase was observed for *PSMB6* (Figure 5B). Inhibition of calpain-1 by PD151746 led to increased levels of uncleaved TCF11/Nrf1 protein, which might explain the modest increase in proteasome subunits.

Finally, the stability of TCF11/Nrf1 after calpain-1/2 inhibition was analysed after inhibition of translation using the translation inhibitor cycloheximide. Here, we found that TCF11/Nrf1 was degraded slower using the inhibitor PD151746, which is more specific for calpain-1 than for calpain-2, compared with untreated cells (Figure 5C).

In summary, this indicates that calpain-1 contributes to degradation TCF11/Nrf1 or at least speed up its degradation by the proteasome under conditions, where the transcription factor is not essentially required for the maintance of proteostasis and cell survival. However, the mechanisms and the biological implications of this interaction remain to be identified.

### Knockout of DDI2 prevents activation of TCF11/Nrf1 and induces faster degradation

**Figure 6:**
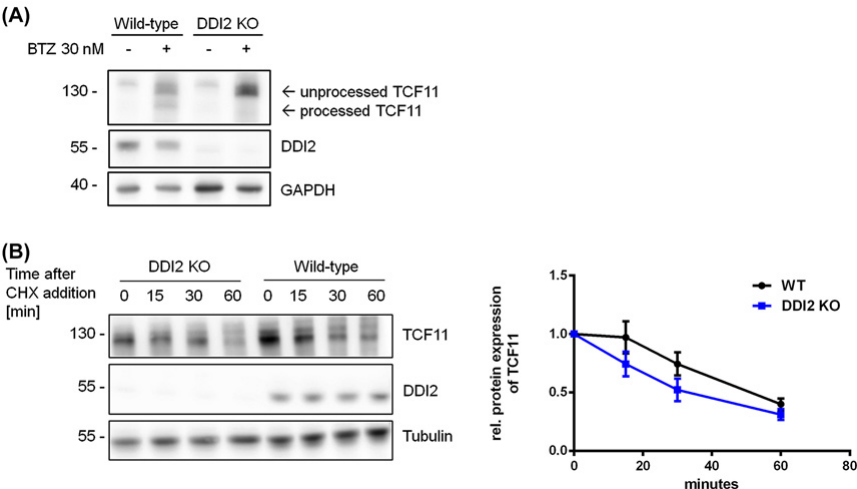
Activation and stability of TCF11/Nrf1 in DDI2 knockout cells (**A**) Ea.Hy-926 cells were treated with 30 nM of the proteasome inhibitor BTZ for 16 h, total cell lysates were analysed by immunoblot, representative figures from *n*=3 each. (**B**) In Ea.Hy-926 cells translation was inhibited with 100 µg/ml cycloheximid. Cells were lysed at indicated time points after adding cyclohexmid and analysed for the expression of TCF11/Nrf1, DDI2 and Tubulin. Bands were analysed by densitometry and normalised to Tubulin and the sample at time point 0, *n*=3.

As it has been published previously that DDI2 is responsible for the activation of TCF11/Nrf1, we wanted to confirm this result in our system. We used CRISPR–Cas to knockout DDI2 which was confirmed using immunoblot analysis. In DDI2 knockout cells, proteasome inhibition using BTZ induced an accumulation of TCF11/Nrf1; however, it was not processed anymore, confirming published results (Figure 6A). Further, we tested the stability of TCF11/Nrf1 in DDI2 knockout cell clones using a cycloheximide chase and found that it was degraded faster in DDI2 knockout cells than in the wild-type cells (Figure 6B). In conclusion, the absence of DDI2 does affect not only TCF11/Nrf1 activation but also its stability.

## Discussion

The transcription factor TCF11/Nrf1 is essential to compensate for loss of proteasomal function by up-regulating the expression of new proteasome subunits in an ERAD-dependent feedback loop. Under non-inducing conditions TCF11/Nrf1 is permanently ubiquitin modified by Hrd1, extracted by p97 and degraded by the proteasome. Under inducing conditions, the ER-membrane bound TCF11/Nrf1 is extracted by p97 and cleaved by DDI2 to translocate into the nucleus [5–10,16]. Although many players have been already identified, the molecular details are only partly understood, and the involvement of other proteases including calpains was controversly discussed.

Here, we show that the protease calpain-1 plays a role in the breakdown of membrane-bound TCF11/Nrf1 but not in the cleavage for activation of TCF11/Nrf1. We provide two lines of evidence for this conclusion. First, we observed that inhibition of calpain-1 had an effect on the stability of the membrane-bound forms of TCF11/Nrf1 as their protein levels were increased when using an inhibitor more specific for calpain-1 and its degradation was delayed. Second, calpain-1 is able to degrade membrane-bound TCF11/Nrf1 *in vitro* even in its mutant form, which is uncleavable for activation in cells. In contrast, we did not see any significant effect of calpain-1 in the context of proteasome inhibition using BTZ in combination with calpain-1/2 inhibitors in different concentrations. Only a modest increase in the protein amount of nuclear TCF11/Nrf1 and mRNA of proteasomal subunits were detected following calpain-1 inhibition and proteasome inhibition. As calpain-1 inhibition enhanced TCF11/Nrf1 protein levels without BTZ present, one might expect that there would also be significant effects following proteasome inhibition. However, it is possible that the increase in TCF11/Nrf1 protein due to calpain-1 inhibition is not high enough to induce significant effects when BTZ is present; especially considering that proteasome inhibition usually only induces a doubling in proteasomal subunit mRNA. Smaller changes in mRNA levels might, therefore, remain undetectable.

Our results partly agree with Zhang et al. [8], who could show that calpain inhibition using CI/ALL, CII and calpeptin had an effect on the activation of promoters of proteasomal subunits and the expression levels of Nrf1. However, they only observed effects with small doses of inhibitor which disappeared when higher doses where used. CI/ALL is not a specific calpain inhibitor as it is also able to inhibit the proteasome and therefore, it remains unclear which inhibiting capability induced the activation of the proteasomal reporters. The other inhibitors, they used, are also not completely specific for calpain and therefore, the observed effects cannot directly be linked to inhibition of calpain-1.

In addition, our data suggest that calpain-1 does not directly affect the activity of TCF11/Nrf1 in the context of proteasome inhibition, but there are other inducers of TCF11/Nrf1 where it might play a role. For example, the inhibitor of complex I of the respiratory chain rotenone has been shown to activate TCF11/Nrf1 [16]. Here, the proteasome is not inhibited but rather more degradation is required as more oxidant-damaged and ubiquitinated proteins accumulate inside the cell. Therefore, it might be possible that other factors play a role in activating TCF11/Nrf1 in different contexts depending on the trigger.

We confirmed that DDI2 is important for the activation of TCF11/Nrf1 when the proteasome is inhibited [7], as knockout of DDI2 by CRISPR–Cas inhibited processing of TCF11/Nrf1. So far, it is unknown whether DDI2 directly cleaves TCF11/Nrf1 or if the interaction is indirect and DDI2 activates another protease, which in turn cleaves TCF11/Nrf1. DDI2 was not present in the membrane fraction during our digestion experiments. This indicates that the cleavage of TCF11/Nrf1 occurs when it already has been pulled out of the membrane by p97 and not when it still resides inside the membrane. We also found that in the absence of DDI2, the degradation of TCF11/Nrf1 is faster. How DDI2 stabilises TCF11/Nrf1 after it has been removed from the membrane is unclear. Within the ERAD-dependent feedback loop of TCF11/Nrf1 degradation and its activating cleavage DDI2 potentially delays the degradation of TCF11/Nrf1 by the proteasome due to its putative ubiquitin binding capacity shown for the yeast homologue Ddi1 [17]. Furthermore, it is also unknown how DDI2 is activated to cleave TCF11/Nrf1 as a previous report showed that purified DDI2 seems to be inactive, either requiring an interacting partner for its cleavage activity or a stimulus to be active [18]. From previous data, it is evident that oxidative stress represents an efficient trigger for activation of TCF11/Nrf1 in addition to proteasomal inhibition [16]. Whether other factors play a role still needs to be determined.

In conclusion, calpain-1 is involved in speeding up the degradation of TCF11/Nrf1 but does not play a direct role in its activation. In this context, one can speculate that calpain-1 action contributes to the degradation pathway of TCF11/Nrf1 under conditions when TCF11/Nrf1 has to be removed quickly from the ER-membrane and/or its extraction by p97 is slow. There are other examples of membrane proteins that require additional proteolytic processing for their final degradation by ERAD [19]. DDI2 in turn delays TCF11/Nrf1 degradation probably by promoting the activation of TCF11/Nrf1 over its degradation pathway.

## Supporting information

**Fig. S1:** Quantification of TCF11/Nrf1 processing after chemical inhibition of calpain-1/2 followed by long-term inhibition of the proteasome

**Fig. S2:** Analysis of TCF11/Nrf1 localisation by immunofluorescence microscopy

**Fig. S3:** Overexpression of CAPN1 and CAPN2 in HEK-293 cells

**Fig. S4:** Test of cleavable and uncleavable mutants of TCF11/Nrf1 in cells and in vitro for calpain degradation.

## Abbreviations

BTZ: bortezomib
CRISPR: clustered regularly interspaced short palindromic repeats
ER: endoplasmic reticulum
ERAD: ER-associated degradation system
DDI2: DNA damage induced-2
HPRT-1: hypoxanthine phosphoribosyltransferase-1
qRT-PCR: quantitative real-time PCR
TCF: transcription factor
UPS: ubiquitin proteasome system
