# Supplementary material for: Inhibition of calpain-1 stabilizes TCF11/Nrf1 but does not affect its activation in response to proteasome inhibition
Source: Biosci Rep. 2018 Sep 19;38(5):BSR20180393. doi: 10.1042/BSR20180393 (PMC6146291; doi:10.1042/BSR20180393)
Supplement: Supplementary file 1 [file bsr20180393_Supp1.pdf]

Fig. S1

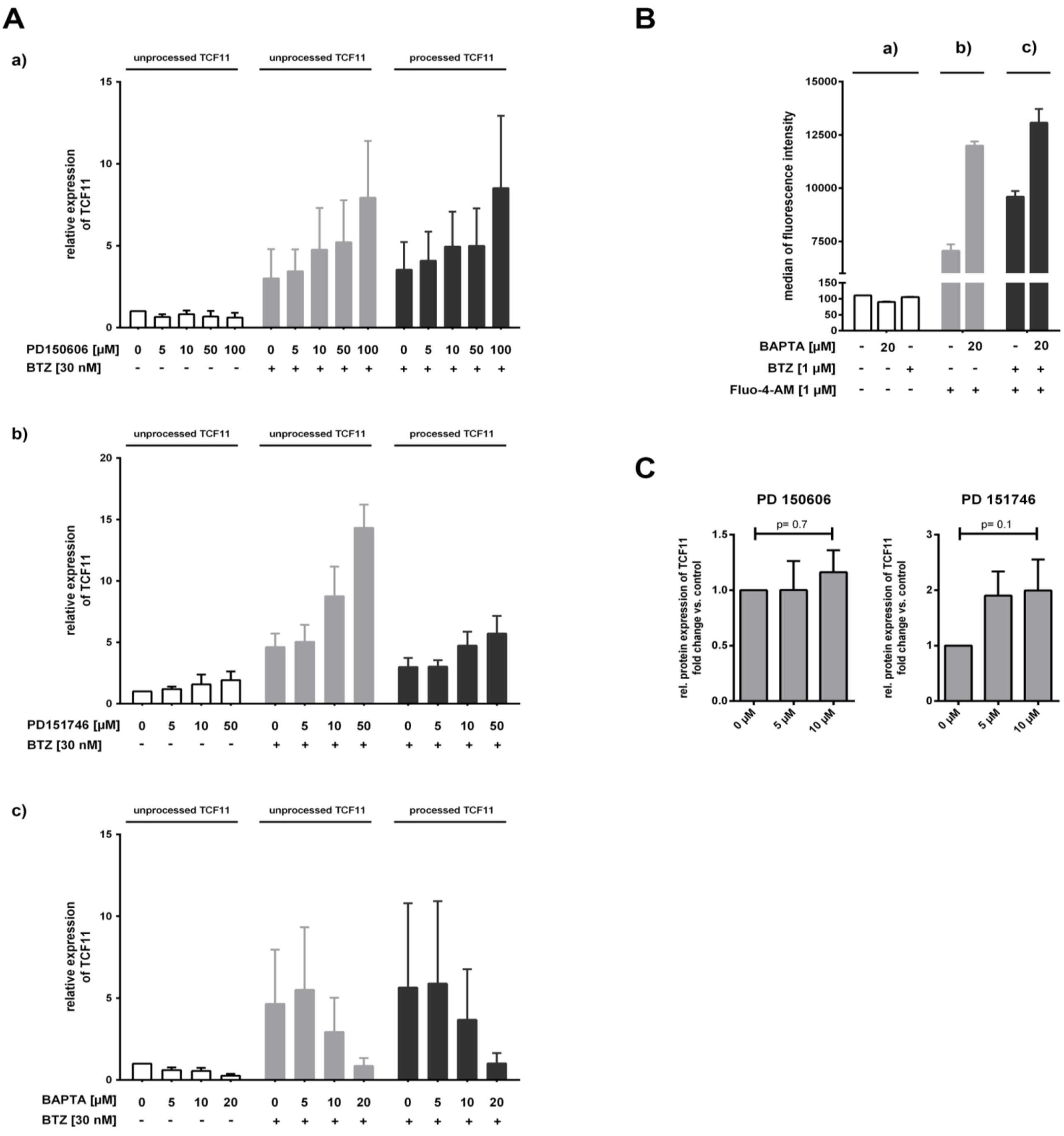
A
a)
unprocessed TCF11
unprocessed TCF11
processed TCF11
relative expression of TCF11
15
10
5
0
PD150606 [µM] 0 5 10 50 100 0 5 10 50 100 0 5 10 50 100
BTZ [30 nM] - - - - - + + + + + + + + + +
b)
unprocessed TCF11
unprocessed TCF11
processed TCF11
relative expression of TCF11
20
15
10
5
0
PD151746 [µM] 0 5 10 50 0 5 10 50 0 5 10 50
BTZ [30 nM] - - - - + + + + + + + +
c)
unprocessed TCF11
unprocessed TCF11
processed TCF11
relative expression of TCF11
15
10
5
0
BAPTA [µM] 0 5 10 20 0 5 10 20 0 5 10 20
BTZ [30 nM] - - - - + + + + + + + +
B
a)
b)
c)
median of fluorescence intensity
15000
12500
10000
7500
150
100
50
0
BAPTA [µM] - 20 - - 20 - 20
BTZ [1 µM] - - + - - + +
Fluo-4-AM [1 µM] - - - + + + +
C
PD 150606
p= 0.7
rel. protein expression of TCF11 fold change vs. control
1.5
1.0
0.5
0.0
0 µM
5 µM
10 µM
PD 151746
p= 0.1
rel. protein expression of TCF11 fold change vs. control
3
2
1
0
0 µM
5 µM
10 µM

Fig. S1: Quantification of TCF11/Nrf1 processing after chemical inhibition of calpain-1/2 followed by long-term inhibition of the proteasome

A: Densitrometric analyses of immunoblots in Fig. 1 (n = 3) were performed and bands normalised to GAPDH. Pixel density of all bands were calculated in relation to the respective control without inhibitors and without BTZ; a) and b) inhibition using specific calpain inhibitors, c) inhibition using the calcium chelator BAPTA-AM.

B: Functional assay for BAPTA-AM; Ea.Hy-926 cells were pretreated for 1 h with BAPTA-AM following BTZ treatment (1 µM) for 3 h. The cells were incubated without/with Fluo-4-AM for 30 min, the calcium-BAPTA complexes maintained in the cell using probenecid, cells were detached by Accutase and used for measurement by flow cytometry. Median fluorescence intensities were given at 525 nm. a) without Fluo-4-AM: control samples for detection of autofluorescence b) with Fluo-4-AM: BAPTA-AM treated samples without proteasomal inhibition by BTZ c) with Fluo-4-AM: BAPTA-AM treated samples with proteasomal inhibition by 1 µM BTZ

C: Influence of the calpain inhibitors for TCF11/Nrf1 protein expression; Ea.Hy-926 cells were treated for 1 h with different inhibitors concentrations. Total cell lysates were analysed by immunoblot for TCF11. Bands were normalised to control, n = 3.

Fig. S2

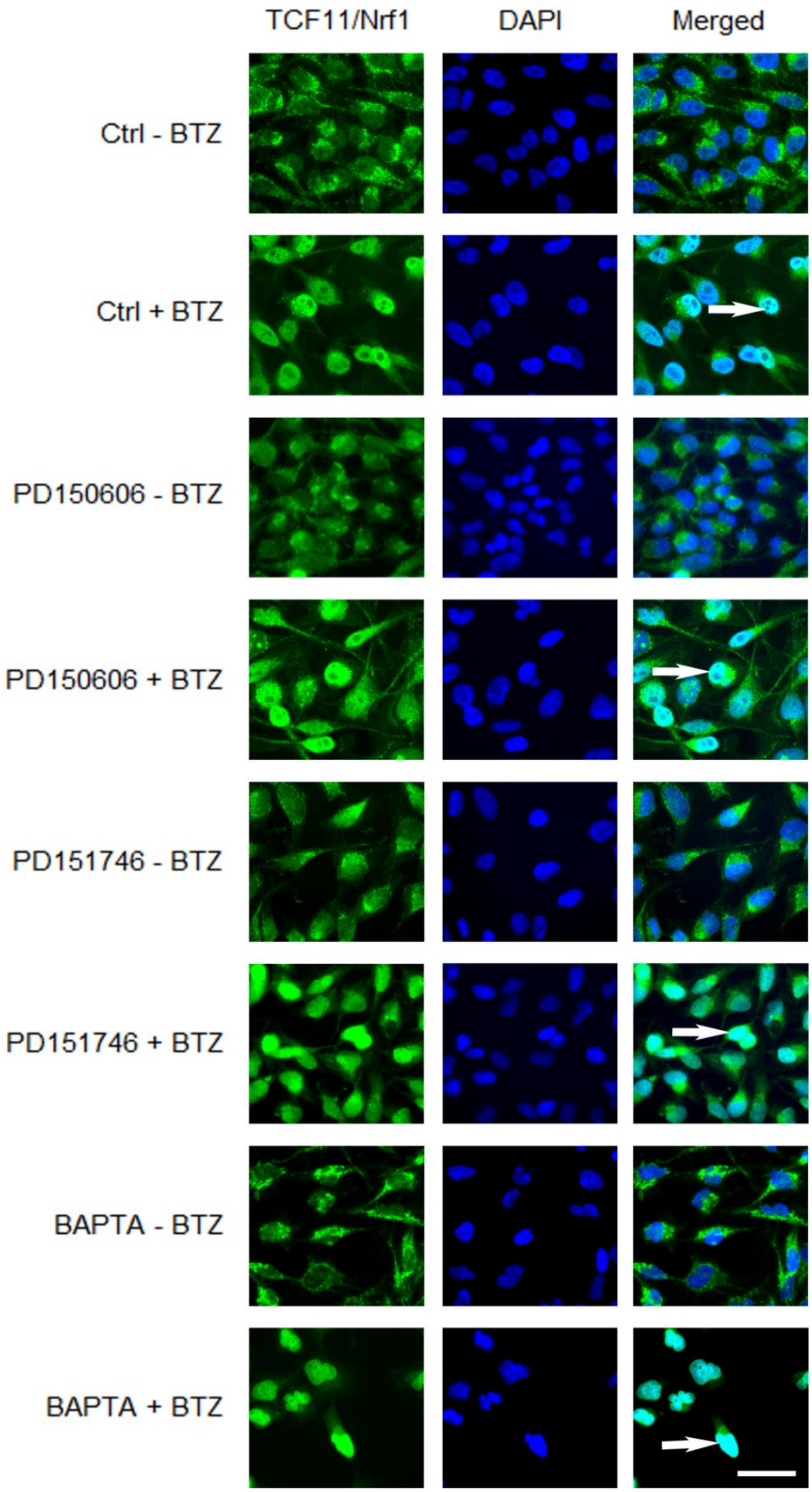

Fig. S2: Analysis of TCF11/Nrf1 localisation by immunofluorescence microscopy
Ea.Hy-926 cells were treated for 1 h with 10 µM of indicated inhibitors and then treated with 30 nM BTZ for 16 h, cells were fixed, permeabilised and stained for TCF11/Nrf1 (green) and DAPI (blue). Scale bar represents 25 µM. Arrows indicate nuclear localisation of TCF11/Nrf1.

Fig. S3

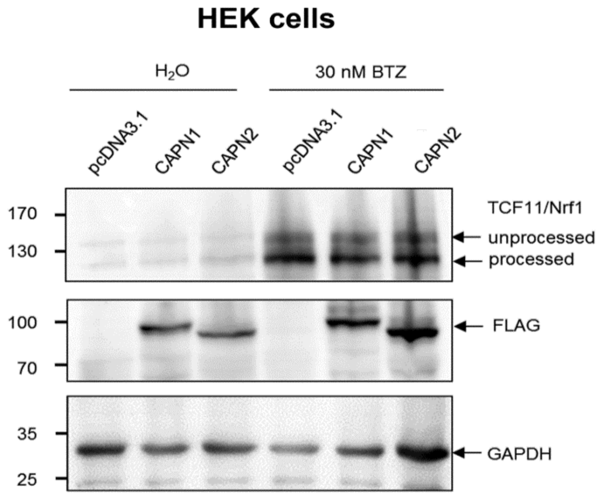

Fig. S3: Overexpression of CAPN1 and CAPN2 in HEK-293 cells

HEK-293 cells were transfected with flag-tagged constructs for the large subunits of calpain-1 (CAPN1) and -2 (CAPN2). The following day, the cells were treated with 30 nM BTZ for 16 hours, total cell lysates were analysed by immunoblot, representative image from n =3.

Fig. S4

A

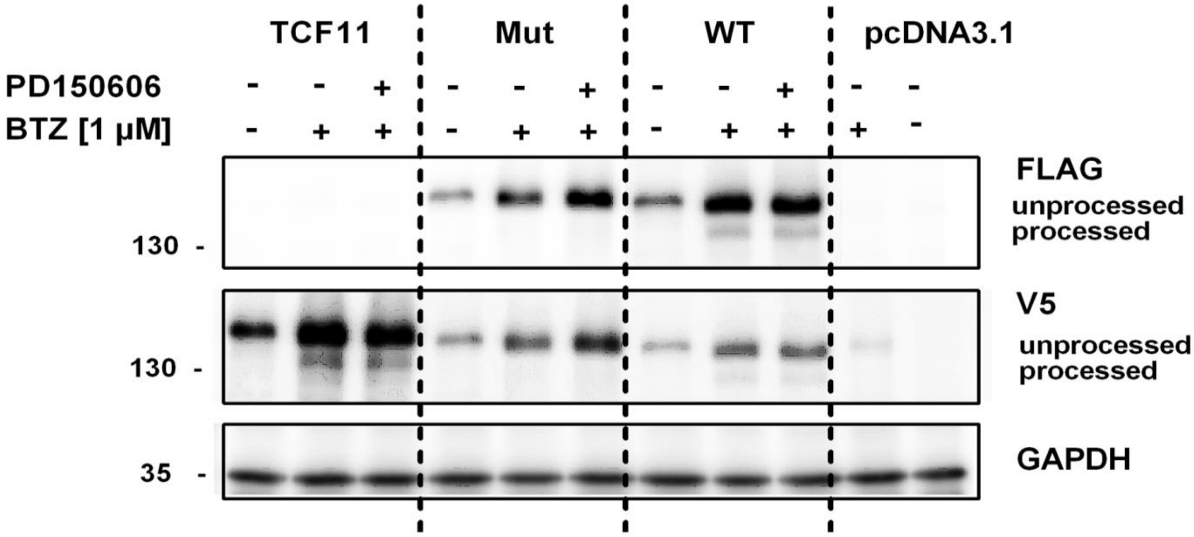
TCF11
Mut
WT
pcDNA3.1
PD150606
BTZ [1 µM]
FLAG
unprocessed
processed
130
V5
unprocessed
processed
130
GAPDH
35

B

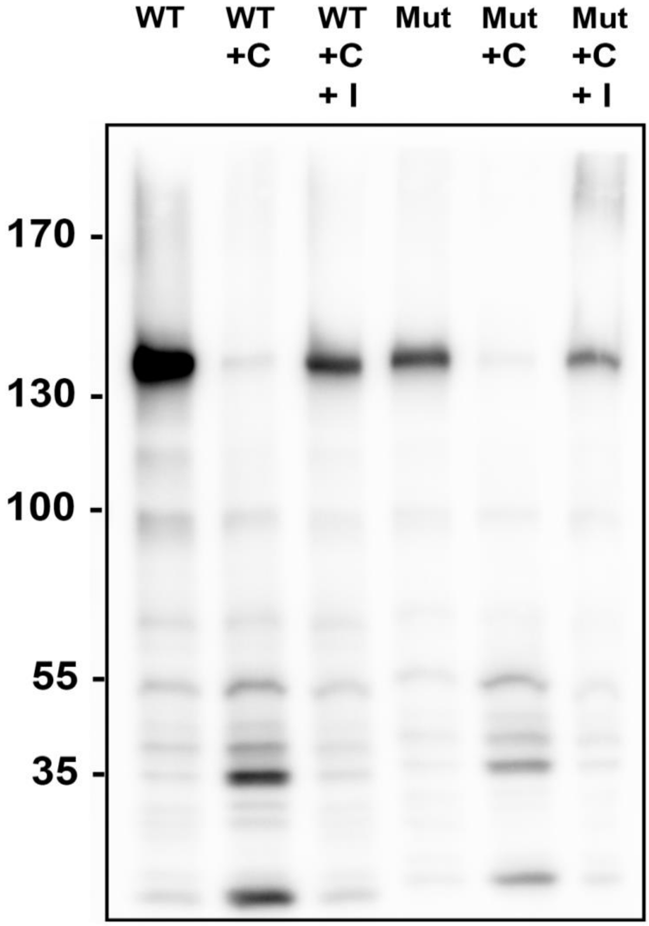
WT
WT +C
WT +C + I
Mut
Mut +C
Mut +C + I
170
130
100
55
35

Fig.S4: Test of cleavable and uncleavable mutants of TCF11/Nrf1 in cells and *in vitro* for calpain degradation.
A: Cells were transfected with TCF11-V5 (TCF11), Wild type-Nrf1 (WT), a mutant Nrf1 version (Mut), which cannot be cleaved anymore, or pcDNA3.1 plasmid. The expression pattern of TCFf11/Nrf1 after proteasome inhibition with 1 µM BTZ were detected. GAPDH was used as a control; n = 3.
B: Digestion of cellular membrane fraction with calpain-1 *in vitro*; Cells were transfected with wild type-Nrf1 (WT) or a mutant Nrf1 version (Mut) which cannot be cleaved anymore. 24 h after transfection, cells were harvested and the membrane fraction was isolated. Calpain reaction buffer, calpain-1(+C) and the impermeant calpain inhibitor Z-LLY-FML (+I) were added to the isolated membrane fraction and incubated for 30 minutes at 37 °C. Samples were analysed by immunoblot for the flag-tag.
